# Neuronal, affective, and sensory correlates of targeted helping behavior in male and female Sprague Dawley rats

**DOI:** 10.3389/fnbeh.2024.1384578

**Published:** 2024-04-10

**Authors:** Stewart S. Cox, Brogan J. Brown, Samuel K. Wood, Samantha J. Brown, Angela M. Kearns, Carmela M. Reichel

**Affiliations:** Department of Neurosciences, Medical University of South Carolina, Charleston, SC, United States

**Keywords:** empathy, *c-fos*, perception action model, sex differences, targeted helping, ultrasonic vocalizations

## Abstract

**Introduction:**

Empathic behaviors are driven by the ability to understand the emotional states of others along with the motivation to improve it. Evidence points towards forms of empathy, like targeted helping, in many species including rats. There are several variables that may modulate targeted helping, including sex, sensory modalities, and activity of multiple neural substrates.

**Methods:**

Using a model of social contact-independent targeted helping, we first tested whether sex differences exist in helping behavior. Next, we explored sex differences in sensory and affective signaling, including direct visualization and an analysis of ultrasonic vocalizations made between animal pairs. Finally, we examined the neural activity in males and females of multiple regions of interest across time. Here, we aim to examine any behavioral differences in our lab’s social contact independent targeted helping task between males and females.

**Results and Discussion:**

These findings are the first to intimate that, like other prosocial behaviors, males and females may exhibit similar social-independent targeted helping behavior, but the underlying sensory communication in males and females may differ. In addition, this is the first set of experiments that explore the neural correlates of social-independent targeted helping in both males and females. These results lay the groundwork for future studies to explore the similarities and differences that drive targeted helping in both sexes.

## Introduction

Empathy is a complex suite of behaviors that works to convey an understanding of the affective states, defined as the underlying experience of emotion, of others. The ability to generate shared affect can have myriad benefits, including group cooperation, reproduction, and survival (Decety, 2011; Preston and de Waal, 2017). Empathic processes, therefore, help to inform interpersonal relationships, as well as guide complex social norms (de Waal and Preston, 2017; Adriaense et al., 2020). A prominent theoretical framework for understanding these behaviors is the Perception Action Model (PAM) (Preston and de Waal, 2002; Decety, 2011; de Waal, 2012; Preston and de Waal, 2017). Complex empathic behaviors are built from, and reliant on, more simplistic ones, at the core is the PAM (Preston and de Waal, 2002; de Waal and Preston, 2017; Preston and de Waal, 2017). According to the PAM, affective transfer occurs between a distressed animal (or “Target”) and a conspecific viewing the Target’s distress (“Observer”), creating a shared affective state (Ben-Ami Bartal et al., 2011; Meyza et al., 2017). The Observer must regulate their new state to perform an action (e.g., targeted helping) to reduce the distress of the Target, and, through a second emotional transfer, themselves (de Waal and Preston, 2017; Cox and Reichel, 2021). This theory has created a cross-species framework to assess empathic processes of varying complexity, with a growing body of evidence indicating numerous mammalian species, including rats, are able to perform prosocial actions such as targeted helping (Preston and de Waal, 2002; Decety, 2011; de Waal, 2012; de Waal and Preston, 2017; Meyza et al., 2017; Preston and de Waal, 2017).

Numerous biological determinants are believed to contribute to the complexity of empathic behavior, including sex. While clinical research has historically shown women have consistently higher levels of empathic concern compared to men (Hall, 1978; O'Brien et al., 2013), these conclusions have recently been called into question (Baez et al., 2017). The few rodent studies using females to explore this question also have conflicting conclusions on the importance of sex in prosocial behaviors. While female rats demonstrated a stronger tendency to release a distressed cagemate compared to males (Ben-Ami Bartal et al., 2011), no sex differences were appreciated when assessing emotional contagion (du et al., 2020; Han et al., 2020). An exploration of the impact sex in a model of targeted helping is therefore necessary to advance the understanding of the field.

According to the PAM, empathic behaviors like targeted helping stem from an affective transfer between conspecifics. Sensory cues are critical for understanding the state of another and can modulate prosocial behaviors, but the mechanism behind this affective transfer is poorly understood and likely multimodal. Research suggests that rodent prosocial behaviors, such as social learning, may require the availability of visual cues for their optimal facilitation (Paraouty et al., 2020). Further, rodents have the ability to recognize the distress of another, at least in part, through visual cues (Nakashima et al., 2015; Geng et al., 2020). Vicarious freezing in an emotional contagion paradigm was attenuated if an Observer’s view of the Target was obstructed by an opaque partition (Geng et al., 2020; Paraouty et al., 2020; Hernandez-Lallement et al., 2022). More recently, it was demonstrated that observational pain contagion in mice requires the image-forming visual system (Geng et al., 2020). We therefore examined the role direct visualization of the distressed conspecific had on Observers during targeted helping in both males and females.

However, it is unlikely that the visual system is the only sensory modality critical in the transfer of emotional information between Observer and Target (Kim et al., 2019; Paraouty et al., 2020; Hernandez-Lallement et al., 2022). Increasing attention has been given to ultrasonic vocalizations (USV) as a proxy for understanding a rodent’s affective state. Although there is still a dearth of evidence regarding the behavioral consequence or specificity of USV in adult rats, they can broadly be categorized into two frequency groups. Low frequency USV, often called 22 kilohertz (kHz) USV, with a range of 18–35 kHz, are emitted in the presence of aversive stimuli, such as a predator or predator odor (Blanchard et al., 1991), and other stressors like inescapable foot shock (Borta et al., 2006). It is hypothesized that low frequency USV serve as alarm calls or indicate an aversive affective state. High frequency USV, also known as 50-kHz USV, fall within the range of >35 kHz and are emitted in prosocial situations like social exploratory activity, mating behavior, and other positive affective states (Panksepp and Burgdorf, 2003; Wohr et al., 2005; Simola and Brudzynski, 2018). The affective valence of rats during targeted helping is not currently known (Chen et al., 2009; Atsak et al., 2011), but some evidence points to USV as critical for evoking a prosocial state and promoting helping behavior (Ben-Ami Bartal et al., 2011). In the following experiments, USV of both the Target and Observer were recorded during multiple timepoints throughout targeted helping in both sexes to understand their respective affective states.

A large library of clinical fMRI studies exists evaluating numerous substrates and their role in empathy. Brain regions that have been correlated with aspects of empathy include those involved in emotional salience and interoceptive valence, specifically the amygdala and insula (Adolphs, 2002; Singer et al., 2004; Fusar-Poli et al., 2009; Keysers and Gazzola, 2018; Marsh, 2018), as well as substrates necessary for perspective-taking, motivation, and cognition, like the prefrontal (PFC), anterior cingulate (ACC), and orbitofrontal (OFC) cortices (Jackson et al., 2005; Singer and Lamm, 2009; Decety, 2015; de Waal and Preston, 2017; Cerniglia et al., 2019; Uysal et al., 2020). More causational and region-specific research using rodent models are beginning to corroborate some of these imaging studies (Adolphs, 2002; Knapska et al., 2006; Panksepp and Panksepp, 2013; Meyza et al., 2017; Meyza and Knapska, 2018; Sivaselvachandran et al., 2018; Carrillo et al., 2019). There has been recent research exploring brain-wide neural activity during helping behavior in male rats (Ben-Ami Bartal et al., 2021), but no work to date has examined sex differences in those regions. Activity of cortical and subcortical regions of interest was evaluated through immunohistochemical analysis of the immediate early gene *c-fos*, an inducible transcription factor important for numerous signaling pathways and a well-established method for identifying changes in neural activity (Bastle et al., 2012). Fos was analyzed across time and between sexes during targeted helping to assess these differences. Further, correlation analysis between *c-fos* activity in each substrate to the latency to aid a conspecific was calculated.

**Figure 1:**
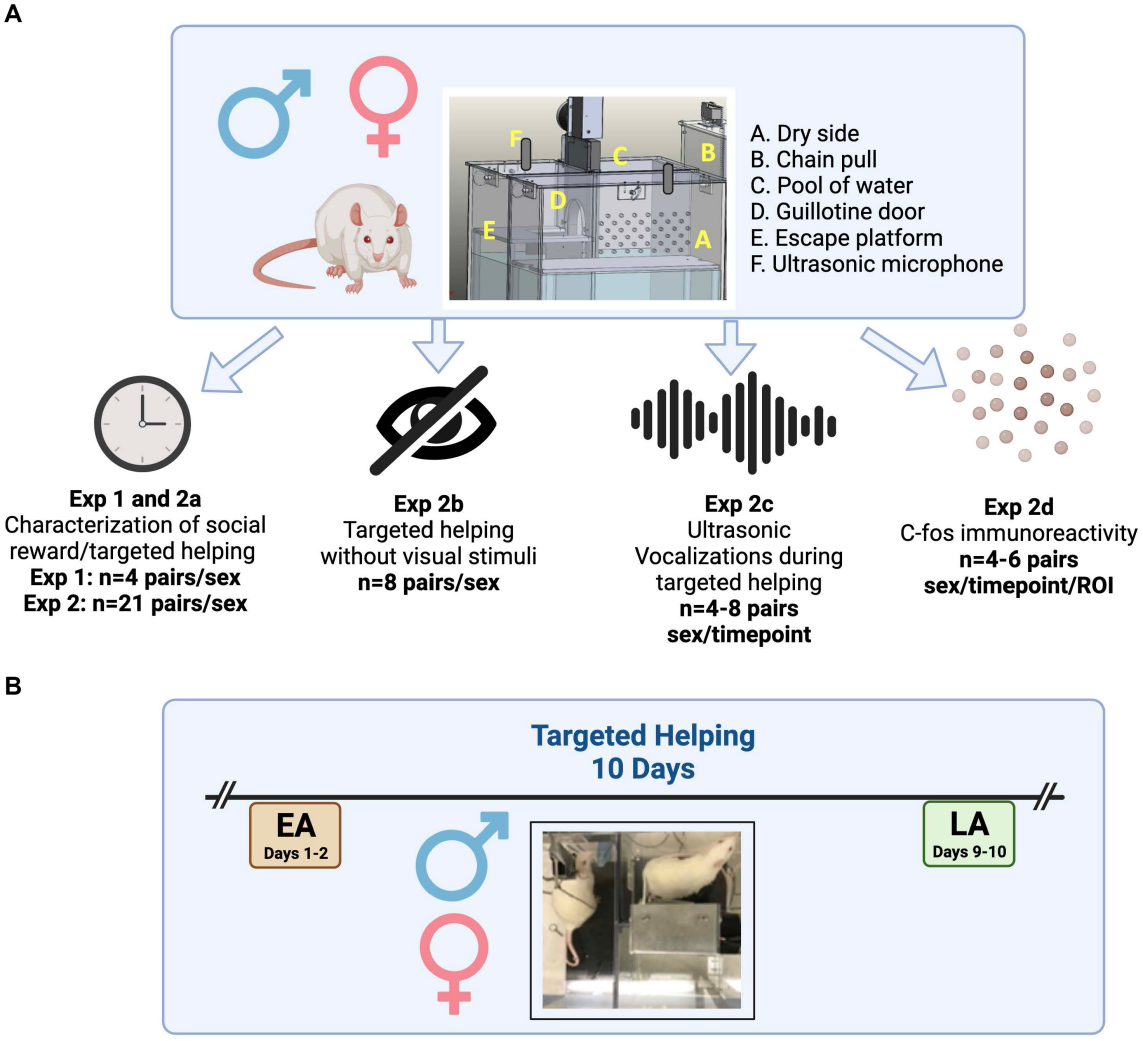
Schematic of variables tested and experimental timeline. A total of 33 pairs of rats/sex were used in Experiments 1–5. **(A)** Size-matched, same-sex males and freely cycling females were used. Social contact-dependent (Exp. 1) or -independent (Exp. 2a) targeted helping was compared between males and females. Further, the impact of direct visualization between Target and Observer (Exp. 2b) and the differences in ultrasonic vocalizations (USV, Exp. 2c) were studied during social contact-independent targeted helping. Finally, subsets of animals were sacrificed, and neural activity within regions of interest was evaluated via immunohistochemistry of the immediate early gene *c-fos* (Exp. 2d). **(B)** Timeline for all behavioral evaluations. One rat in a cage pair was randomly assigned to be the Target or Observer. During acquisition, Observers were placed in the dry side of the chamber and given the opportunity to release the Target from the water chamber. Data were collected during three timepoints: early acquisition (EA, orange), as an indication of an initial helping response; and late acquisition (LA, green) to examine the effect of habituation to aiding a familiar conspecific.

In Experiment 2, we used a model of social contact-independent targeted helping, in which one animal (Targets) is placed in a compartment of the apparatus filled with 100 mm of water. The other animal (Observers) was placed on a dry platform with access to a chain that, when pulled, opens an automated door that releases the Target into a dry compartment separate from the Observer (Cox and Reichel, 2020). Chain pulling in this task does not appear to be merely a consequence of a learned motor response, as previous work shows an inconsistent trend in chain pulling when a conspecific is present in the apparatus but not in a distressed condition (Cox and Reichel, 2020). Other studies examining other motor outputs in targeted helping tasks have similar outcomes (Ben-Ami Bartal et al., 2011; Sato et al., 2015; Ben-Ami Bartal et al., 2016); moreover, our model also removes social contact as a possible rewarding confound for the observed outcome (Cox and Reichel, 2020; Cox et al., 2022). We used this model to investigate the behavioral, affective, sensory, and neural differences in targeted helping between same-sex conspecifics in both males and females across two timepoints; early acquisition (EA), which was used as an indication of an initial helping response; and late acquisition (LA), a timepoint used to examine whether additional experience with releasing a familiar conspecific modulates helping behavior as it does social interaction (Choleris et al., 2006; Sato et al., 2015; Yamagishi et al., 2020) (see Figure 1 for schematic representation of experiments and the experimental timeline). We did not find any significant sex differences in the timepoints examined. While the behavioral outcome is similar, it is likely that targeted helping may have sex-specific neural and affective mechanisms. This set of experiments work to elucidate some of the underlying sex differences in sensory communication and changes in neural activity that mediate the behavior evaluated in this targeted helping task.

## Methods

### Animals

Size-matched male and female Sprague Dawley rats (*n* = 33 pairs/sex total) weighing 250–275 g (8–10 weeks of age at start of experiment) were pair-housed with the same sex on a 12-h reversed light cycle (lights on at 1800). The breakdown of animals used is visualized in Figure 1. Animals were given food and water *ad libitum* until behavioral testing when they were then switched to a daily stable intake (20 g) of rat chow (Harlan) as a more ethologically-relevant feeding protocol used in our laboratory and recommended by the Universities Federation for Animal Welfare (UFAW) (Savenije et al., 2010). Rats were given at least 5 days to acclimate to their cagemate (Savenije et al., 2010; Cox and Reichel, 2020; Cox et al., 2022). Following acclimation, one rat was randomly selected to be the “Observer” and the other the “Target.” Animals were also acclimated to the transportation process and the experiment room, and handled/weighed for 2 days, 5 min/day before the behavioral assessment. For all behavioral evaluations, rats were transported to the experiment room and left undisturbed for 5 min. The tasks were performed during the rats dark cycle in a sound-attenuated room with the lights off except for a single lamp at the opposite end of the room used for the experimenter to view the test. The testing apparatus was cleaned following each trial. All experimental procedures were conducted in accordance with the “Guide for the Care and Use of Laboratory Rats” (Institute of Laboratory Animal Resources on Life Sciences, National Research Council) and approved by The Institutional Animal Care and Use Committee (IACUC) of the Medical University of South Carolina.

### Behavioral testing

#### Experiment 1: social contact-dependent release task

Evaluation of social contact-dependent release behavior was performed in males and females (*n* = 4 pairs/sex) using a custom-made operant box (34.2 × 33.9 × 30.5 cm) by Med Associates (Fairfax, VT, United States; see Cox and Reichel, 2020 for schematic). Targets were placed in 100 mm of water in the wet compartment of the apparatus, while the Observer was placed on a dry platform with access to a chain that, when pulled, opened an automatic guillotine door. Door opening allowed Targets to be released into the same dry compartment as the Observer (Cox and Reichel, 2020).

#### Experiment 2: social contact-independent targeted helping task

In all of studies in Experiment 2, targeted helping behavior was evaluated (*n* = 29pairs/sex) using a custom (Med Associates; Fairfax, VT, United States) operant box developed with three chambers (Cox and Reichel, 2020, 2021; Cox et al., 2022), as seen in Figure 1A. In this apparatus, Targets were placed in 100 mm of water in the wet compartment and Observers on a dry platform with access to a chain that opened an automated door. The Target was released into a dry compartment separate from the Observer (Exp. 2a, *n* = 21 pairs/sex). Throughout Experiment 2, latency to chain pull was taken as an index of helping behavior (Ben-Ami Bartal et al., 2011; Cox and Reichel, 2020; Cox et al., 2022). Trials (20 total across 10 days, labeled “Acquisition”) lasted a total of 300 s (5 min) regardless of the chain pull latency. For all behavioral assessments, two trials were conducted daily during the rats’ dark cycle (Cox and Reichel, 2020; Cox et al., 2022). If the Observer did not pull the chain within the allotted time, the experimenter ended the trial and released the Target. We focused on two timepoints for analysis (see Figure 1): early acquisition (EA, average of days 1–2), as an indication of an initial helping response; and late acquisition (LA, average of days 9–10) to examine the effect of habituation to aiding a familiar conspecific. In order to determine the importance of the Observer visualizing the Target to learn to release the distressed conspecific, a separate cohort of male and female rats (Exp. 2b, *n* = 8 pairs/sex) was tested in the same operant box, but the Plexiglas divider present between Observer and Target was painted black to prevent either animal from seeing through it.

### Ultrasonic vocalization detection and analysis

For Exp. 2c, ultrasonic vocalizations (USV) were recorded during targeted helping at EA and LA in a subset of male and female rats (*n* = 4–8 groups/sex/timepoint), also used for behavioral and Fos analysis (Exps. 2a and 2d), to understand their affective states and level of communication during targeted helping. Two high-quality condenser microphones (Avisoft Bioacoustics) were fastened to the lids of the operant box, one on the Observer’s dry side and one on the Target’s wet side. The microphones were connected to Avisoft UltraSoundGate 416Hb multichannel recording system and processed using Avisoft-SASLab Pro software (Avisoft Bioacoustics, Glienicke, Germany). USV were recorded for one complete trial (300 s) at both timepoints (EA and LA) with a sampling rate of 250 kHz and analyzed with DeepSqueak version 2.6.0 (Coffey et al., 2019) in MATLAB. Due to background noise during the task, *post-hoc* denoising was carried out and subsequently rechecked for errors by an experimenter. Calls with tonality of <0.35 were considered to be background and were rejected manually from analysis. Remaining USV were reviewed by an experimenter blind to the conditions and calls that were picked up in both microphones were assigned to a particular rat by comparing power and timing of each USV across both channels. USV data (Exp. 2c) were binned in 5 kHz increments and relative frequencies (% of total) were calculated for each timepoint to assess distribution of calls across frequency spectrum. USV of each rat were categorized within the two broad categories “distress” (18–35 kHz) or ‘prosocial’ (>35 kHz) and analyzed with two-way between subjects ANOVAs for total call counts, as well as percent of total distress prosocial calls, with sex and group as the variables.

### Immunohistochemistry

In Exp. 2d, a subset of rats (*n* = 4–6/sex/timepoint) were sacrificed to evaluate *c-fos* activity as a consequence of targeted helping in several regions of interests. At each timepoint (EA and LA), Observers either performed the targeted helping task (Behaving, BEH) or remained in their homecages as controls (HCC). Importantly, HCCs had the same behavioral history as the BEH rats, except they did not perform the task on test day. These rats were sacrificed and perfused approximately 90 min following targeted helping and brains were collected as has been described thoroughly in manuscripts published from our lab and others (Bobadilla et al., 2017; Peters et al., 2018; Cox et al., 2022; Carter et al., 2023). Briefly, rats were anesthetized with Equithesin and then transcardially perfused with 150–200 mL cold 0.9% saline followed by 400–500 mL of 10% buffered formalin. Brains were removed and postfixed in 10% formalin for 24 h, submerged in 20% sucrose/0.1% sodium azide solution for 48 h, and then sectioned into 50 μm tissue sections. For Fos expression, tissue sections were incubated in a rabbit anti-Fos primary antibody (Millipore; 1:1000; RRID 310107) overnight, followed by a 2-h incubation in donkey anti-rabbit secondary antibody (Jackson ImmunoResearch; 1:500; RRID 2340584) amplified with an avidin biotin complex method (Thermo Scientific, Waltham, MA). The sections were then visualized with 3,3′ diaminobenzidine (DAB, Sigma-Aldrich, St. Louis, MO) + nickel ammonium sulfate to produce a blue-black nuclear reaction product. Slices were coverslipped using Permount, and regions of interest were photographed at 10× magnification using a Leica microscope and VideoToolbox software.

#### IHC quantification and analysis

For the DAB stain, blue-black nuclear immunoprecipitate from Fos-positive cells (Fos+) in regions of interest were quantified using a brain atlas for comparison (Paxinos et al., 1980). Anterior–posterior coordinates for each analyzed region are as follows: AI: 3.5 to 1.5; PL: 3.7 to 2.7; IL: 3.2 to 2.2; ACC: 3.2 to 2.2; OFC: 4.2 to 2.7; LHb: −2.5 to −3.3; PVT: −2.5 to −3.6; BLA: −2.5 to −3.3; CeA: −2.5 to −2.8. Regions of Interest were utilized using a brain atlas for comparison and standardized across all animals evaluated. These regions were photographed at 10× magnification using a Leica microscope and VideoToolbox software. All images were quantified using ImageJ software (NIH) by an experimenter blinded to the conditions. Fos+ cells that fell within each region of interest were counted using a macro and averaged across sections for each rat. On average, 3 bilateral sequential sections for each region were used for analysis. In order to compare the change in Fos+ cells between sex and across time, each group was also compared to their own homecage control (HCC) by calculating a percent change from HCC in the analysis (see Supplementary Figure S2). Animals were excluded from analysis of a particular brain region if 3 sequential sections were not available.

### Data analysis

Two-way mixed analyses of variance (ANOVAs) were used to compare the latency of chain pulls responses for social contact-dependent release behavior (Exp. 1) and targeted helping (Exp. 2a) as well as targeted helping when vision is obstructed (Exp. 2b). The between-subject variable was sex (males vs. females), with the repeated measure being days (1–10). Comparison of the timepoints defined as EA (acquisition days 1–2) and LA (acquisition days 9–10) was performed with unpaired *t*-test between males and females. USV data (Exp. 2c) were binned in 5 kHz increments, and relative frequencies (% of total) were calculated for each timepoint. USV of each rat were categorized as “distress” (18–35 kHz) or “prosocial” (>35 kHz) and analyzed with two-way between subjects ANOVAs for total call counts, as well as percent of total distress prosocial calls, with sex and group as the variables. Three-way mixed variable ANOVAs, in order to account for any missing samples, were used to analyze total Fos + cell counts (Exp. 2d) during EA and LA with brain region, sex, and group as the independent variables. When correlations were calculated, total Fos count was correlated with release latency at all three timepoints separately using a Spearman R correlational analysis. All *post hoc* comparisons were conducted using a Holm-Sidak’s correction for family wise error when appropriate, with the alpha set at 0.05. Mixed effect models were used when necessary to account for any missing data points. All analyses were conducted with Prism Software version 9.0. Unless noted, all data are expressed as the mean ± SEM.

## Results

### Experiment 1. Males and females readily release a conspecific when social interaction is possible

**Figure 2:**
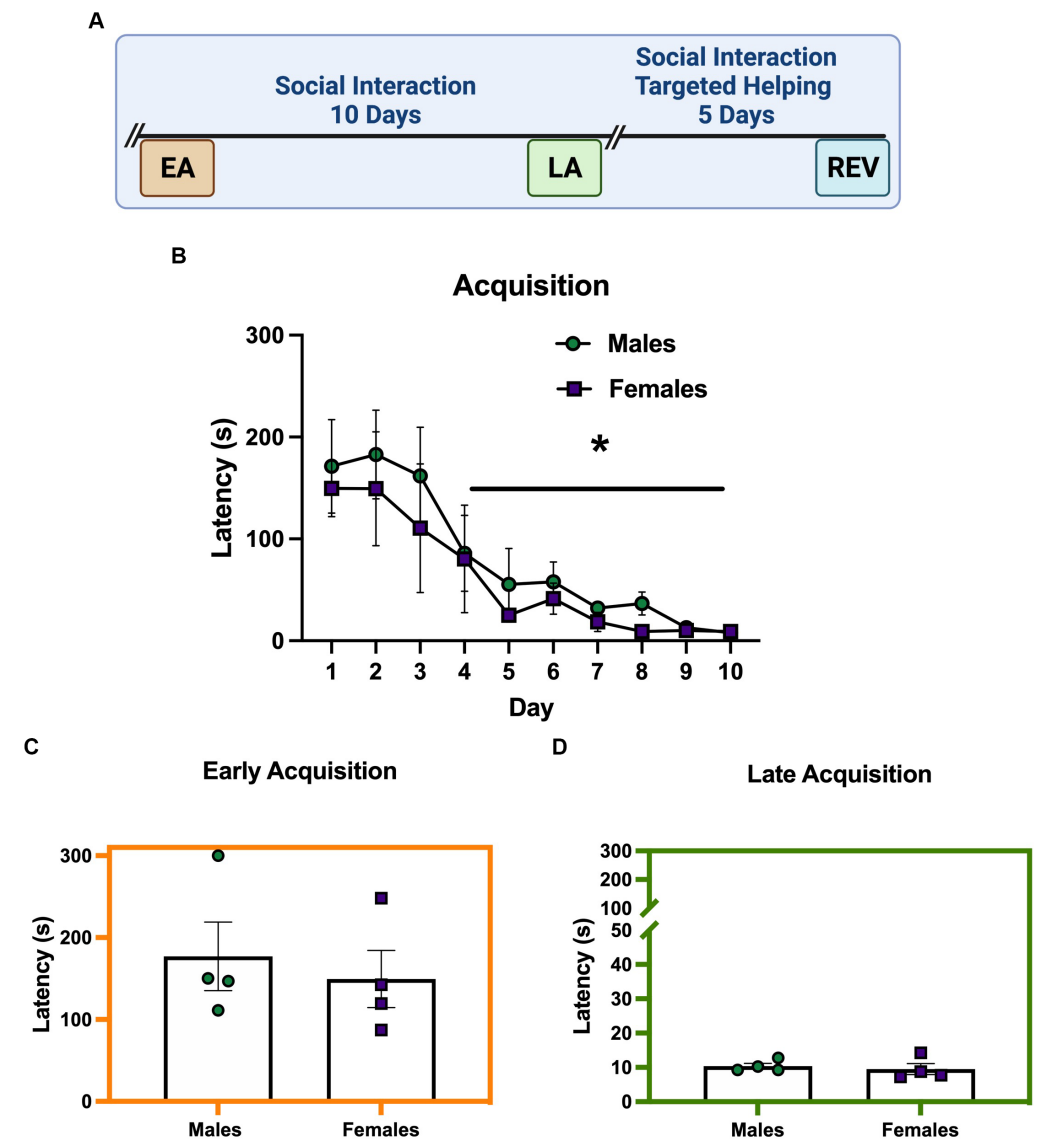
Males and females readily release a conspecific when social interaction is possible. **(A)** Performance of male and female (*n* = 4 pairs/sex) rats during the helping task where social contact is possible. **(B)** Chain pull latencies for males and females during acquisition did not differ; latencies decreased over time, with days 4–10 significantly faster compared to day 1. **(C,D)** Unpaired *t*-tests comparing chain pull latencies during early (EA) **(C)** and late (LA) **(D)** acquisition did not show a difference between males and females. Error bars represent ± SEM. *Significant difference from day 1, *p* < 0.05.

Figure 2A depicts the timeline for males and females performing a release task that allows for social interaction. In this task, a chain pull response released the Target into the same chamber as the Observer (Cox and Reichel, 2020). Figure 2B demonstrates that, during acquisition, males and females (*n* = 4/sex) release a distressed conspecific in a targeted helping apparatus that affords social contact at similar rates (main effect of time [*F* (9, 54) =10.03, *p* < 0.0001]). *Post hoc* analysis of the main effect showed chain pull latency on days 4–10 was significantly faster compared to day 1 (*p* < 0.05), similar to what our lab has shown previously (Cox and Reichel, 2020). The 2-way ANOVA did not reveal a main effect of sex during acquisition. Indeed, no differences were seen in the unpaired *t*-tests comparing male and female latencies during EA (Figure 2C) or LA (Figure 2D).

### Experiment 2a. Targeted helping in male and female rats

**Figure 3:**
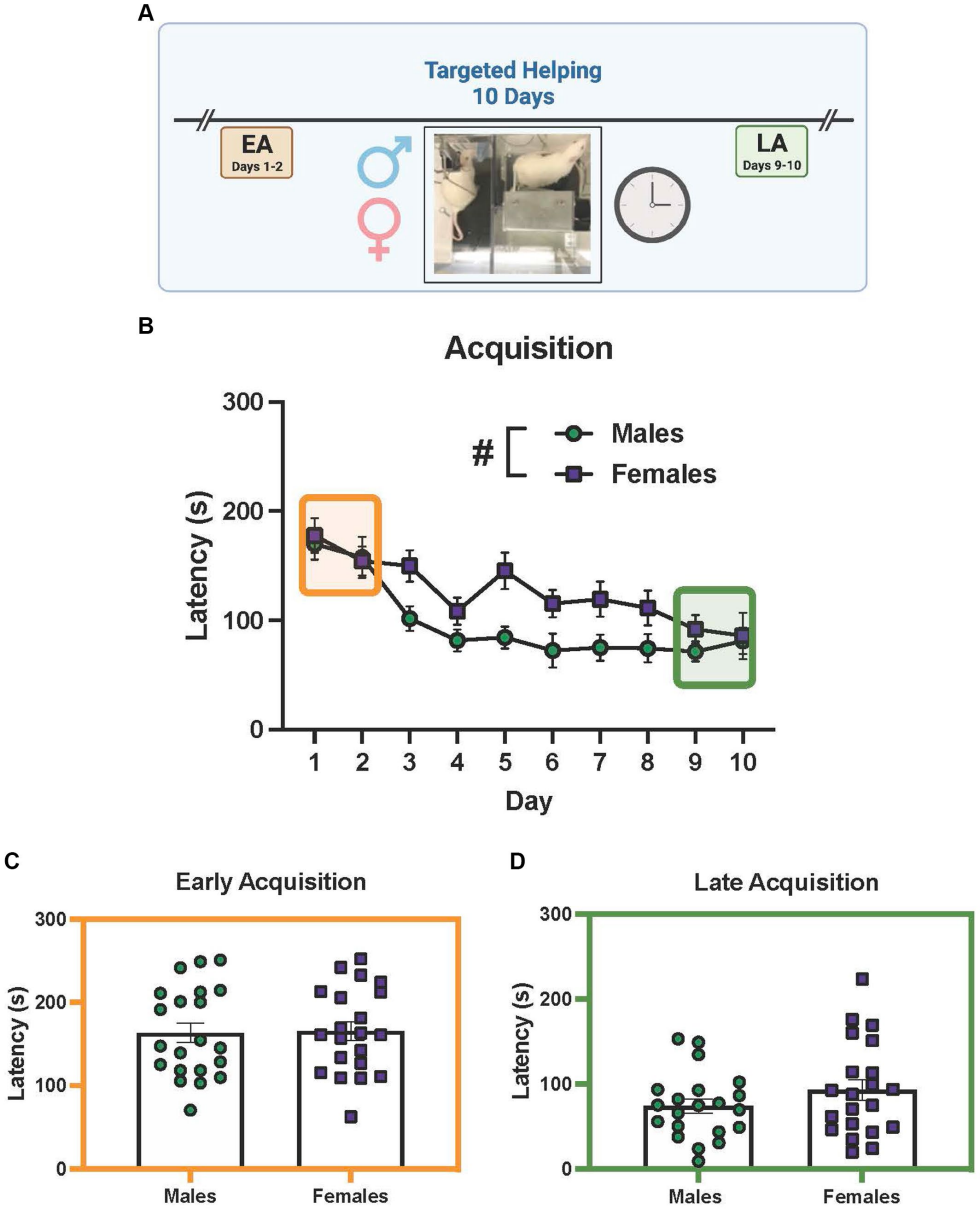
Elucidation of sex differences during targeted helping. **(A)** Performance of male and female (*n* = 21 pairs/sex) rats during the social contact-independent targeted helping task revealed the Observers’ latency to release a distressed partner decreased over 10 days. Significantly shorter latencies occurred on days 3–10 compared to day 1. **(B)** A main effect of sex was also seen in acquisition, with male latencies being faster compared to females. **(C,D)** Unpaired *t*-tests comparing chain pull latencies during early (EA) **(C)** and late (LA) **(D)** acquisition did not show a difference between males and females. Error bars represent ± SEM.*Significant difference from day 1, *p* < 0.05. #Significant difference between males and females, *p* < 0.05.

Groups of male and female Observers (*n* = 21 pairs/sex) performed our lab’s social contact-independent targeted helping task (Cox and Reichel, 2020; Cox et al., 2022) to discern if any sex differences in chain pull latency were present (Figure 3A depicts the timeline). For the acquisition phase, a mixed effects 2-way ANOVA showed a main effect of time [Figure 3B, *F*(9, 344) = 13.00, *p* < 0.0001] and sex [*F* (1, 40) = 7.272, *p* = 0.0102], with males having faster chain pull latencies compared to females. However, the time × sex interaction was not significant. *Post hoc* analysis on the main effect of time revealed the latencies on days 2–10 were significantly faster (*p* < 0.0005) compared to day 1 (Figure 2B). In order to determine if sex differences were present during the timepoints assessed [early (EA) and late (LA) acquisition], averages were taken for latencies on days 1–2 (EA) and 9–10 (LA) for each sex and subsequently compared (Figures 3C,D). Unpaired *t*-tests showed no significant difference between males and females at either timepoint.

### Experiment 2b. Sex differences in targeted helping when visualization of the conspecific is prevented

**Figure 4:**
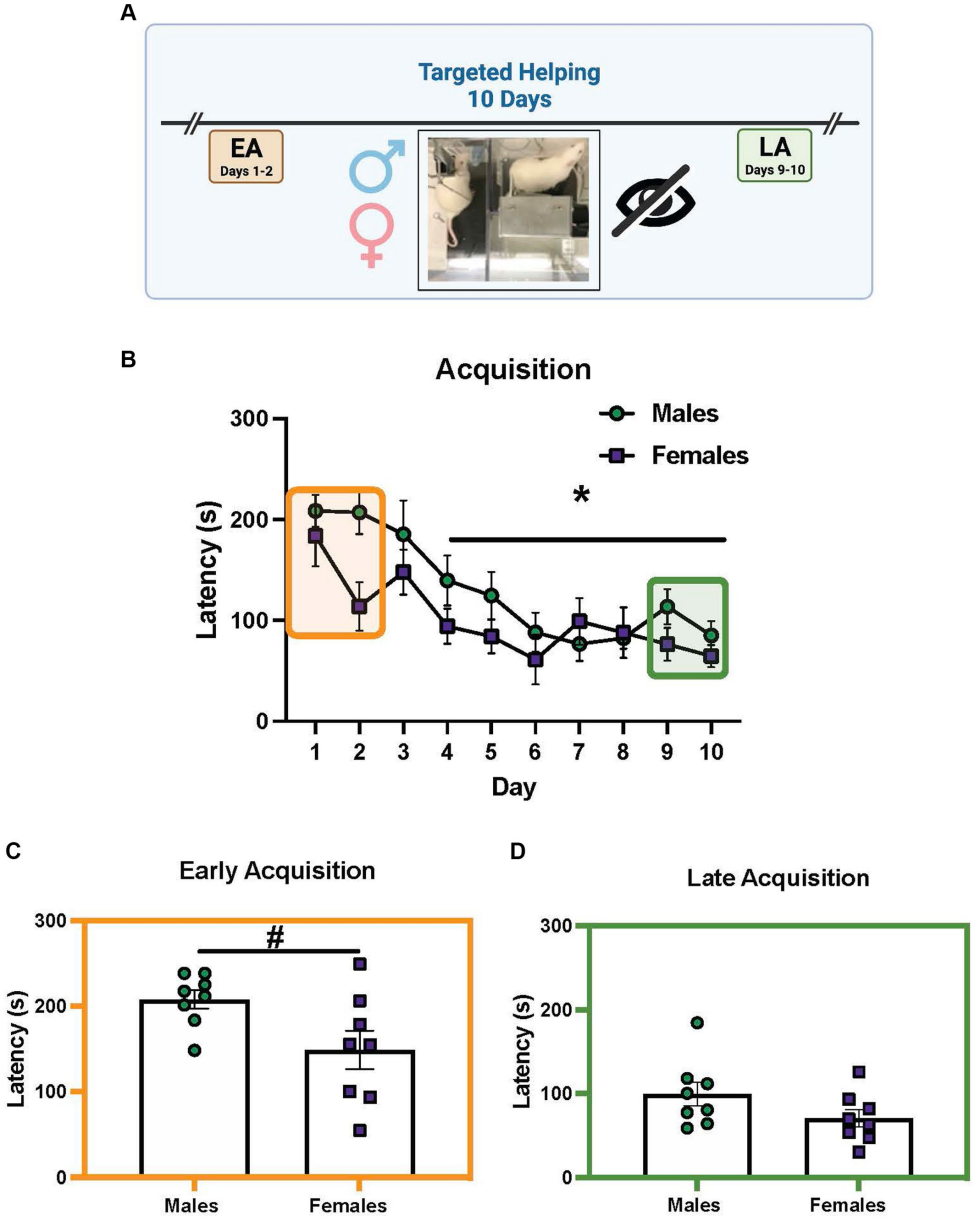
Sex differences in helping behavior when visualization of conspecific is prevented. **(A)** Performance of male and female (*n* = 8 pairs/sex) rats during the social contact-independent targeted helping task when sight of the Target was obstructed. **(B)** Male and female chain pull latencies decreased over time, with days 4–10 significantly faster compared to day 1. There was a strong trend toward a main effect of sex, but it did not reach significance (*p* = 0.0589). **(C,D)** Unpaired *t*-tests were performed comparing the average latencies for early (EA) and late (LA) acquisition between males and females. **(B)** Female latencies were significantly faster compared to males in EA. **(D)** During LA, however, there was no sex difference in chain pull latency. Error bars represent ± SEM. *Significant difference from day 1, *p* < 0.05. #Significant difference between males and females, *p* < 0.05.

Figure 4 shows the chain pull latency of males and females (*n* = 8 pairs/sex) during targeted helping when the Plexiglas divider between the Observer and Target was obstructed. Only a main effect of time [*F* (9, 126) = 9.733, *p* < 0.0001] was revealed by the 2-way ANOVA during acquisition, with days 4–10 significantly different from day 1. There was a strong trend for an effect of sex [*F* (1, 14) = 4.27, *p* = 0.0589, Figure 4A]. Unpaired *t*-tests comparing males and females during EA and LA revealed females were significantly faster during EA compared to males [Figure 4B, *T*(14) = 2.36, *p* = 0.043], but not during LA (Figure 4C).

### Experiment 2c. Ultrasonic vocalizations during targeted helping

**Figure 5:**
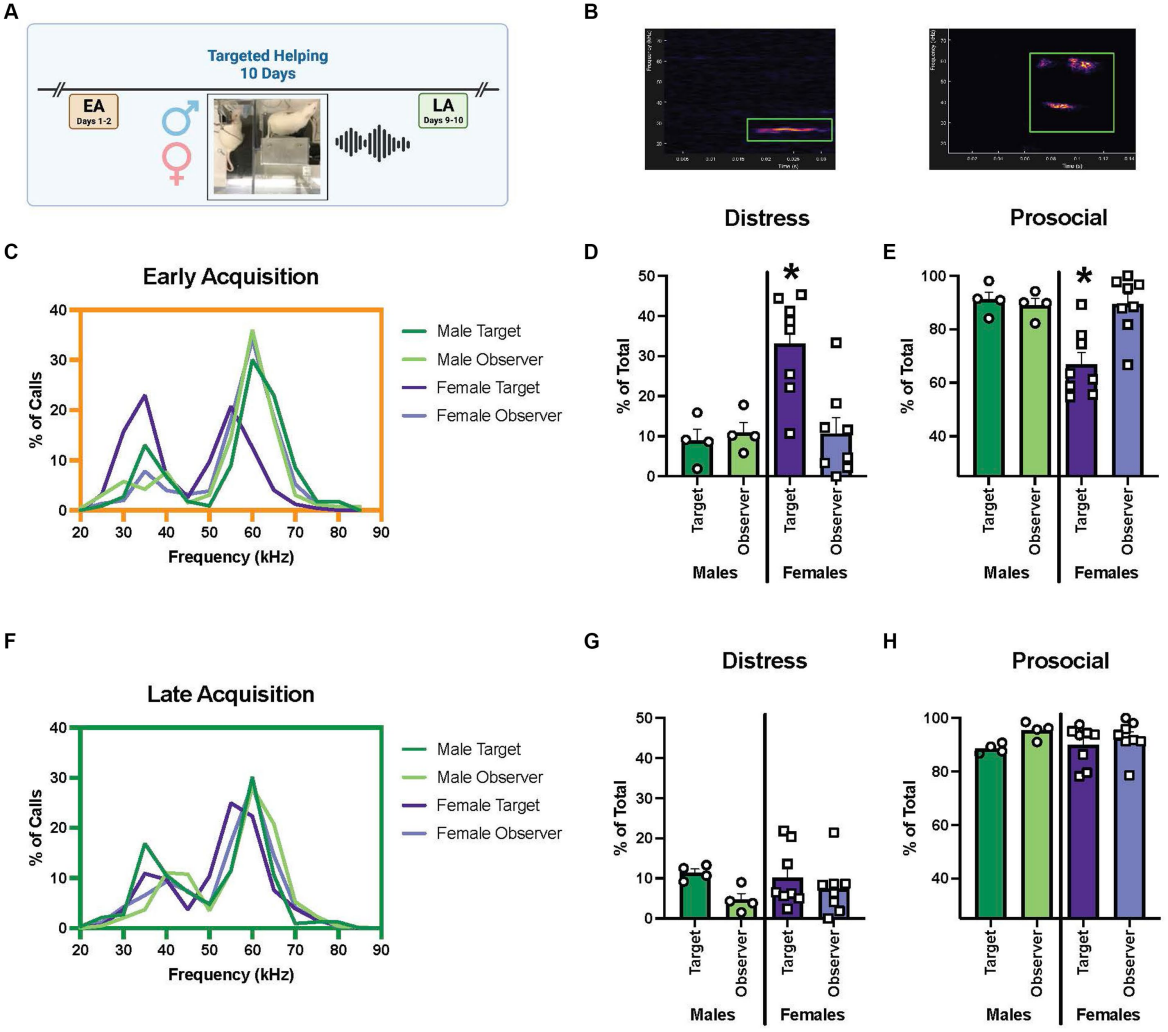
Comparison of USV frequencies during targeted helping between males and females. **(A)** Experimental timeline for helping behavior. Ultrasonic vocalizations (USV) were recorded during early (EA) and late (LA) acquisition, as well as reversal (Rev) as a proxy for the rats’ affective state during the task. USV were recorded and categorized based on frequency into two groups, “distress” (18–35 kHz) or “prosocial” (>35 kHz). **(B)** An example of a distress and prosocial call shown in DeepSqueak software. **(C–E)** USV analysis for early acquisition. **(C)** Frequency of distribution graph of all calls during EA indicates a bimodal distribution of call frequencies, roughly corresponding to the “distress” and “prosocial” ranges, in all groups. **(D,E)** An analysis of calls broken into distress or prosocial range for each group as measured by a percentage of total calls made. **(D)** Female Targets make a significantly greater percentage of distress calls in EA compared to all other groups. **(E)** Correspondingly, calls in the prosocial range make up a significantly smaller percentage of female Targets’ total calls compared to the other groups. **(F–H)** USV analysis during late acquisition. **(F)** Bimodal distribution of calls is seen in frequency of distribution graph of all calls during LA, however, fewer calls are made in the distress range. **(G,H)** No differences were seen between groups in % of calls made in distress **(G)** or prosocial **(H)** ranges. Error bars represent ± SEM. *p < 0.05.

In this experiment, a subset of male and female rats (*n* = 4–8 groups/sex) went through the targeted helping task (Exp. 2a), and ultrasonic vocalizations (USV) at the timepoints of interest (EA, LA) were recorded (see Figure 5A). In adult rats, two main USV have been categorized; aversive (between 18 and 35 kHz) calls during stressful events, and prosocial/appetitive (>35 kHz) calls (Wohr et al., 2005; Wohr and Schwarting, 2007; Takahashi et al., 2010; Wohr et al., 2016; Cox and Reichel, 2021), are used broadly to understand rats’ affect. Samples of each of these calls evaluated using DeepSqueak (Coffey et al., 2019) are depicted in Figure 5B. To determine the range and proportion of communicative frequencies during the task in males and females, calls for each sex in EA and LA were used to generate a distribution of frequencies graph of the call frequencies (kHz). Call frequencies were binned in 5 kHz increments, and relative frequencies (% of total) were calculated for each timepoint. USV of each rat were categorized as “distress” (18–35 kHz) or “prosocial” (>35 kHz), and each category was analyzed as a percent of total calls using 2-way ANOVAs, with sex (male vs. female) and group (Targets vs. Observers) as the variables.

During EA, the frequency distribution analysis indicated a bimodal distribution of call frequencies (Figure 5C). When the calls were split into “distress” or “prosocial,” there were main effects of sex [*F* (1, 20) = 7.33, *p* = 0.0136], group [*F* (1, 20) = 5.278, *p* = 0.0325], and a sex × group interaction [*F* (1, 20) = 7.655, *p* = 0.0119]. *Post hoc* analysis indicated female Targets had a significantly larger proportion of their total calls fall within the distress range, and therefore significantly fewer within the prosocial range, compared to all other groups at EA (*p* < 0.005) (Figures 5D,E). The same analysis was performed for males and females at the LA timepoint; a bimodal distribution with a more limited number of vocalizations in the distress range was seen (Figure 5F). In fact, no difference in the percent of distress or prosocial calls in male or female Observers or Targets was found (Figures 5G,H).

### Experiment 2d: *c-fos* total count varies across substrate, group, and sex during targeted helping

In order to discern the neural correlates of targeted helping, as well as the temporal changes and sex effects on these neural substrates, a subset of male and female Observers that were used in Experiment 2a (*n* = 4–6/sex/region of interest) were sacrificed to evaluate *c-fos* activity in multiple regions of interest. At the timepoints of interest (EA, LA), rats either performed targeted helping (Behaving, BEH), or remained in their homecages as controls (HCC). Importantly, HCC had the same behavioral history as the BEH rats, except they did not perform the task on test day. The total number of Fos + cells within each region of interest was quantified (see Methods).

**Figure 6:**
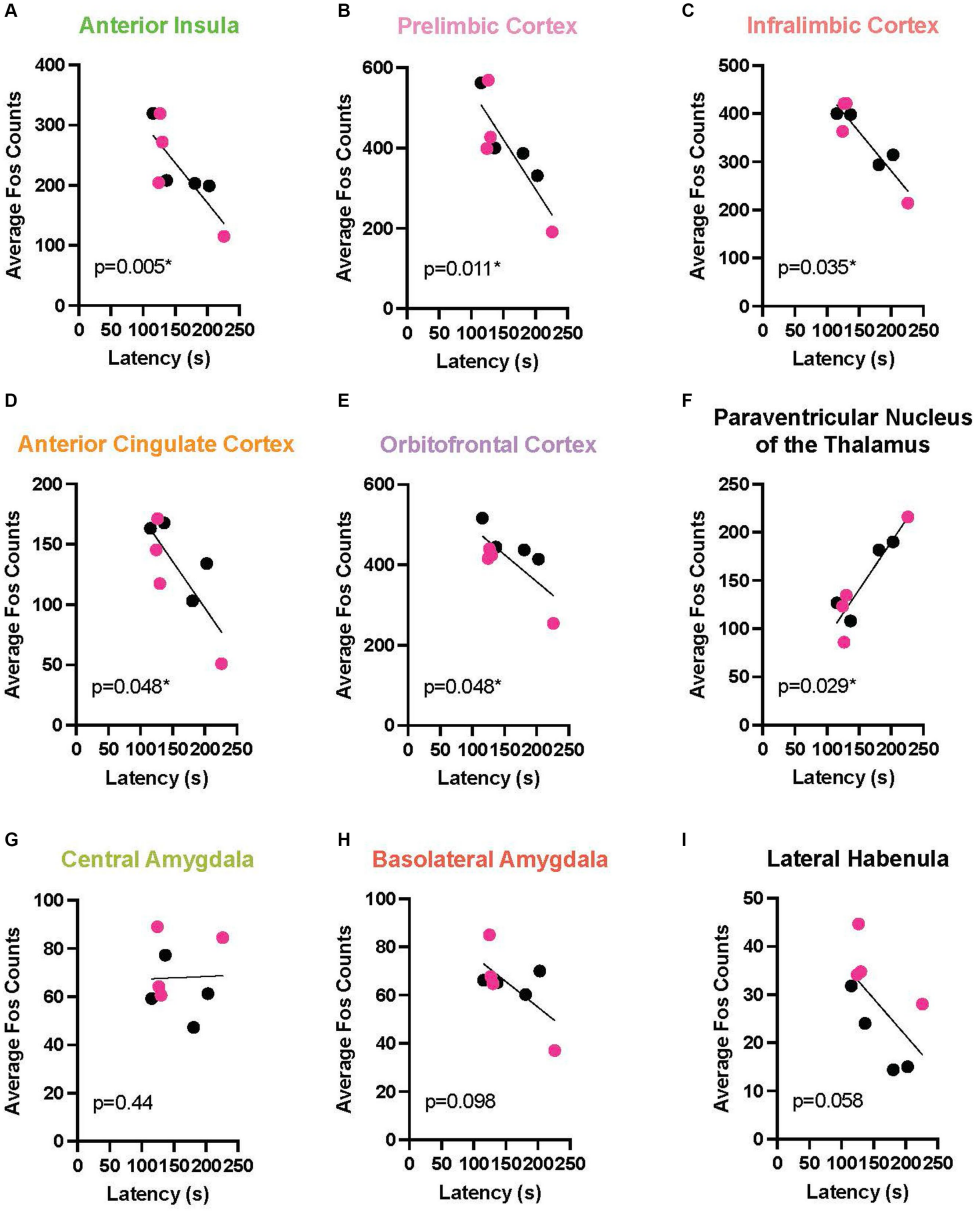
Correlation between release latency and Fos activity during early acquisition. In order to determine a relationship between helping behavior and a neurobiological endpoint, correlational analysis was performed between the last latency and average Fos + cell counts for male (data points in black) and female (red) Observers during early acquisition in the following regions of interest: anterior insula **(A)**, prelimbic **(B)**, infralimbic **(C)**, anterior cingulate **(D)**, and orbitofrontal **(E)** cortices, paraventricular nucleus of the thalamus (PVT) **(F)**, central amygdala **(G)**, basolateral amygdala **(H)**, and lateral habenula **(I)**. Significant negative correlations were found between final latency and mean Fos count in the five cortical regions analyzed. In contrast, a positive correlation was observed in the PVT. *Significant correlation, *p* < 0.05.

**Figure 7:**
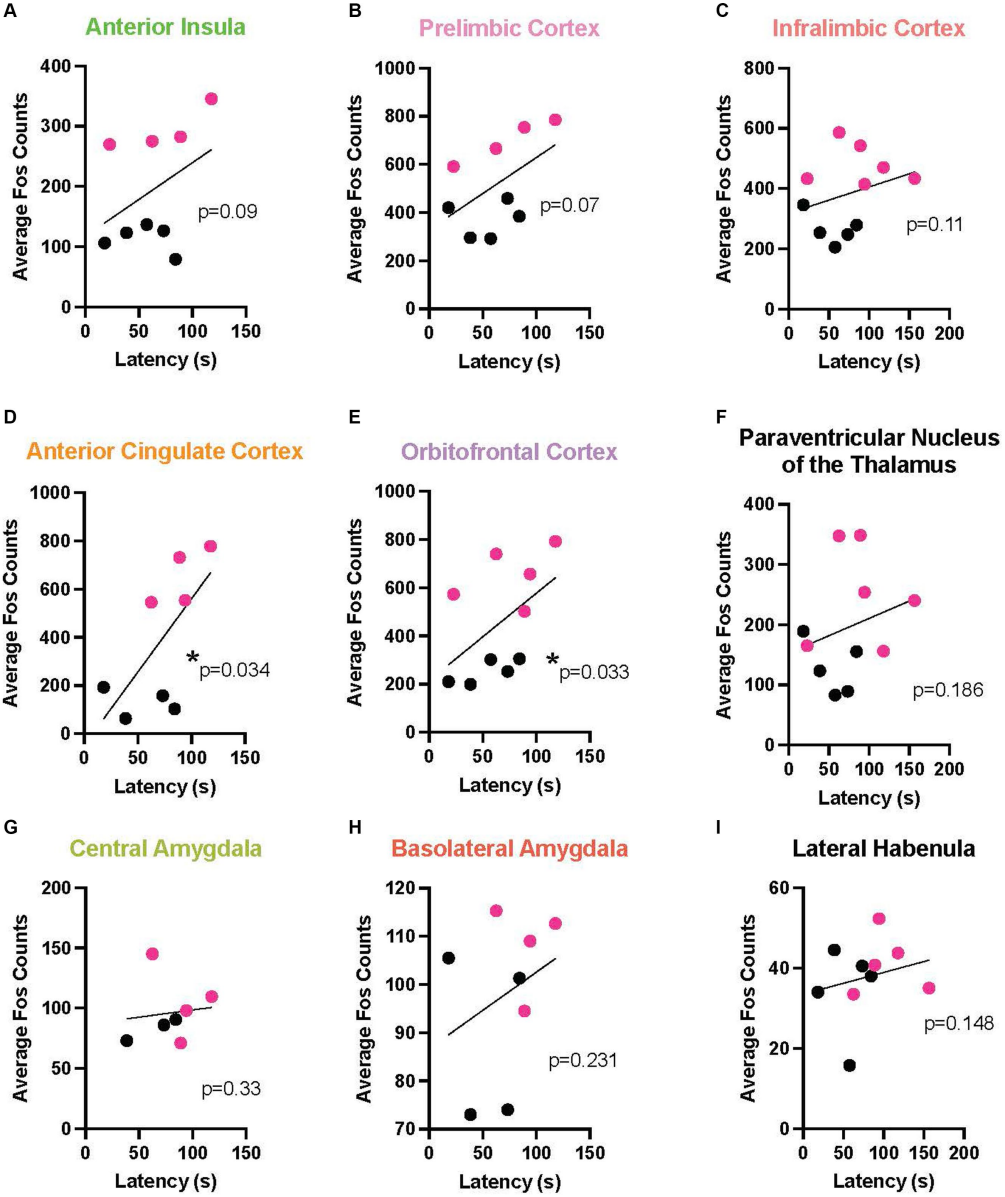
Correlation between release latency and Fos activity during late acquisition. In order to determine a relationship between helping behavior and a neurobiological endpoint, correlational analysis was performed between the last latency and average Fos + cell counts for male (data points in black) and female (red) Observers during late acquisition in the following regions of interest: anterior insula **(A)**, prelimbic **(B)**, infralimbic **(C)**, anterior cingulate **(D)**, and orbitofrontal **(E)** cortices, paraventricular nucleus of the thalamus (PVT) **(F)**, central amygdala **(G)**, basolateral amygdala **(H)**, and lateral habenula **(I)**. Significant positive correlations were found in the anterior cingulate and orbitofrontal cortices, with strong trends also seen in the anterior insula and prelimbic cortex. *Significant correlation, *p* < 0.05.

To link the behavior with a neurobiological endpoint, we determined the relationship between chain pull latency and neural activation across multiple brain areas during EA and LA. In this analysis, because no sex differences were found at any timepoint, males and females were combined. In EA, a marked negative relationship emerged between Fos activation and response latency in multiple cortical areas, including: the AI (*r* = 0.857, *p* < 0.0054), the PL (*r* = 0.809, *p* < 0.0109), IL (*r* = 0.690, *p* < 0.0347), ACC (*r* = 0.643, *p* < 0.048), and OFC (*r* = 0.649, *p* < 0.0481). In these areas, longer response latencies were associated with reduced neural activity levels as measured by Fos + cells. A positive relationship also emerged in the PVT (*r* = 0.714, *p* < 0.0288), meaning as latencies increased, so did neural activity (Figure 6 depicts these correlations). During LA (Figure 7), there were positive correlations, rather than negative, reaching significance in the ACC (*r* = 0.690, *p* < 0.0347) and the OFC (*r* = 0.612, *p* < 0.0334).

**Figure 8:**
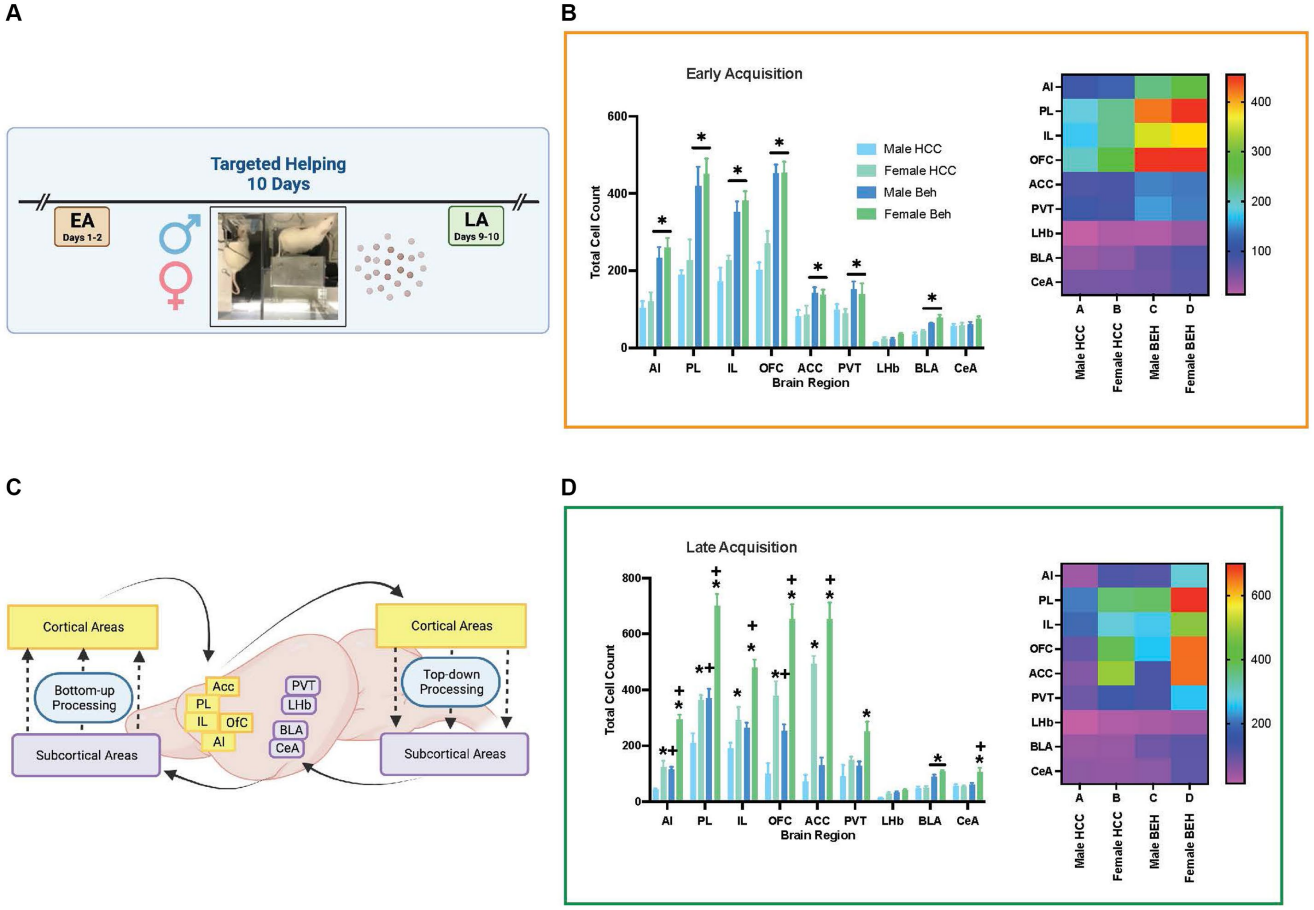
Differences in total Fos + cells across neural substrates of interest. Total Fos + cells were quantified in male and female rats that performed the targeted helping task (Behaving, BEH) or were left in their homecage on test day (HCC). Rats (4–6 groups/sex) were sacrificed at two different timepoints; the second day of acquisition (EA), or the final day of acquisition (LA). Fos + counts were compared in each timepoint. **(A)** This figure depicts total Fos + cell counts for male and female HCC and Beh rats during EA. **(B)** Representative heat map depicting Fos + cell means in each region of interest for each group. **(C,D)** Fos activity in neural substrates during LA **(C)**, with the heat map depicting mean activity in each region across the four groups **(D)**. Error bars represent ± SEM. *Significant difference between males and females, *p* < 0.05. +Significant difference between BEH and HCC within the same sex, *p* < 0.05.

Next, each timepoint was analyzed as a 3-way mixed ANOVA with sex (male, female), group (HCC, BEH), and brain region as the variables. At EA (Figure 8B), there was a brain region × group interaction [*F* (8, 96) = 19.56, *p* < 0.0001] as well as main effects of brain region [*F* (8, 96) = 152.6, *p* < 0.0001] and group [*F* (1, 13) = 69.42, *p* < 0.0001]. There were no other main effects or interactions. *Post hoc* comparisons reveal that behaving animals had greater cellular activity in the AI, PL, IL, OFC, ACC, PVT, and BLA (*p*’s = 0.047–0.0001). A heat map (Figure 8B) depicts this interaction by presenting the mean of each substrate during EA.

At LA (Figure 8D), there were three significant 2-way interactions: brain region × group [*F* (8, 107) = 9.85, *p* < 0.0001]; brain region × sex [*F* (8, 107) = 38.15, *p* < 0.0001]; and group × sex [*F* (8, 16) = 8.29, *p* < 0.0113] as well as main effects of brain region [*F* (8, 107) = 125.1, *p* < 0.0001], group [*F* (1, 6) = 64.61, *p* < 0.0001], and sex [*F* (1, 16) = 145.8, *p* < 0.0001]. To decompose these interactions, we conducted the analysis for each brain area separately. In the AI, there was a significant group × sex interaction [*F* (1, 13) = 10.77, *p* < 0.006], and a *post hoc* comparison shows greater AI Fos activation in females relative to males in both HCC (*p* < 0.0075) and BEH (*p* < 0.0001) rats. Also, greater activation was seen in BEH rats relative to HCC rats in both males (*p* < 0.008) and females (*p* < 0.0001). In the PL, there was a significant group × sex interaction [*F* (1, 13) = 6.54, *p* < 0.024] and *post hoc* comparisons show greater AI Fos activation in females relative to males in both HCC (*p* < 0.018) and BEH (*p* < 0.0001) rats, as well as greater activation in BEH relative to HCC rats in both males (*p* < 0.0.014) and females (*p* < 0.0001). In the IL, there were main effects of group [*F* (1, 16) = 19.71, *p* < 0.0004] and sex [*F* (1, 16) = 28.9, *p* < 0.0001]. There were main effects of group [*F* (1, 14) = 24.71, *p* < 0.0002] and sex [*F* (1, 14) = 61.99, *p* < 0.0001] found in the OFC. In the ACC there were main effects of group [*F* (1, 12) = 24.71, *p* < 0.015] and sex [*F* (1, 12) = 153, *p* < 0.0001]. Main effects of group [*F* (1, 16) = 5.81, *p* < 0.028] and sex [*F* (1, 16) = 9.68, *p* < 0.007] were also found in the PVT. In the LHb there were main effects of group [*F* (1, 15) = 11.28, *p* < 0.0043] and sex [*F* (1, 15) = 7.25, *p* < 0.02]. Only a main effect of group [*F* (1, 12) = 53.26, *p* < 0.0001] was seen in the BLA. Finally, in the CeA, there was a significant group × sex interaction [*F* (1, 12) = 7.23, *p* < 0.02]. *Post hoc* comparison showed greater Fos activation in female BEH relative to HCC (*p* < 0.0085) and female relative to male BEH (*p* < 0.0001) rats. A heat map (Figure 8D) depicts this interaction by presenting the mean of each region during LA.

Noting the sex differences in HCC animals that were evident across cortical and subcortical areas, we highlight these baseline differences in Supplementary Figure S1. To account for relative changes in neural activation, Fos + cell counts were adjusted to a percent of baseline and analyzed over the different timepoints (Supplementary Figure S2).

## Discussion

In the current set of experiments, we sought to elucidate behavioral, sensory, affective, and neural variables of targeted helping in male and female rats. Overall, males and females do not exhibit any differences in the drive for social reward (Experiment 1, Figure 2). Additionally, no difference in release latency was found between males and females at the timepoints analyzed during targeted helping (Experiment 2a, Figure 3). However, we found that similar behavioral outcomes between sexes may be driven by different mechanisms or socio-emotional cues. For example, females exhibited faster latency to release the distressed target when direct visualization was blocked in early acquisition (EA) (Figure 4). Further, female Targets made significantly more ultrasonic vocalizations (USV) in the distress/aversive frequencies (Figure 5) compared to males.

In addition, a change in the relationship of the correlation between cortical activity and release latency across time in acquisition was found across both sexes, suggesting a level of plasticity in cortical neurons over the course of targeted helping (Figures 6, 7). Interestingly, when assessing male and female neural activity separately, disparate neural patterns were exhibited during late acquisition (LA), but similar patterns in early acquisition (EA) (Figure 8).

The broadly held assumption that females exhibit higher levels of empathic concern compared to males (Hall, 1978; O'Brien et al., 2013) has not held up to experimental scrutiny in clinical experiments (Eisenberg and Lennon, 1983; Baez et al., 2017). The few rodent studies exploring sex differences in emotional contagion or prosocial behavior also show mixed results; targeted helping is potentiated in females compared to males in some studies (Ben-Ami Bartal et al., 2011), while other studies using emotional contagion report no difference (du et al., 2020; Han et al., 2020). Our studies indicate males and females do not seem to exhibit a difference in the motivation for social reward, as latencies were similar between sexes in the model of targeted helping that affords social interaction (Figure 2) (Cox and Reichel, 2020; Silva et al., 2020). In a social contact-independent model of targeted helping, we also found no sex differences in the timepoints analyzed during acquisition (EA, LA; Figure 3), suggesting similar levels of helping behavior between sexes. However, it is possible other unmeasured physiologic variables also contribute to release latency. For example, evidence suggests female rats demonstrate potentiated anxiety-like behaviors and corticosterone levels, proposed to play a role in helping behavior, compared to males (Ben-Ami Bartal et al., 2016; Karakilic et al., 2018; Scholl et al., 2019). This difference in anxiety-like behaviors may be directly related to a heightened affective transfer in females or associated with cycling hormonal levels (Mora et al., 1996; Mikosz et al., 2015). Additionally, the interaction of reproductive hormones over the estrous cycle has not been established in targeted helping models. Research using emotional contagion as a proxy of empathic concern showed females in the diestral phase of the estrous cycle behaved similarly to males but were significantly less responsive during the estrus phase (Mora et al., 1996). While these questions were outside the scope of this set of experiments, the notion that circulating hormonal changes can contribute to helping behaviors do warrant further study. Future work could compare changes in corticosterone levels in males and females as an additional biologic marker for stress level across timepoints, as it is possible Targets may have acclimated to the distressing condition over time. Further, assessing ovariectomized females compared to freely cycling females could help elucidate the impact of estrogens on helping behavior. In addition, throughout these experiments cagemates were used as dyads for every trial, meaning each Observer was familiar with their respective Target. Our lab and others have demonstrated Observers can overcome the initial attenuation of release latency with additional trials (Cox and Reichel, 2020), can make in-group generalizations based on prior experience (Ben-Ami Bartal et al., 2011), and may be driven by different hormonal signals depending on their familiarity with the Target (Martin et al., 2015). Future studies analyzing the impact of familiarity on prosocial behaviors across sex would be of great value to the field.

As mentioned previously, evidence indicates complex, situational, and multimodal sensory communication is required to necessitate affective transfer and targeted helping (Meyza et al., 2017; Kim et al., 2019; Hernandez-Lallement et al., 2022). Females released the Target faster than males when direct visualization was obstructed at EA (Figures 4A,B). We propose females maintain similar latencies even if sight is obstructed, while males may rely more heavily on visualization of the Target early in acquisition. In addition, during EA, female Targets emit a higher proportion of distress USV compared to male Targets based (Figure 5). These USV data expand on the findings in the literature that show Targets made more stress calls early in acquisition when release was rare (Ben-Ami Bartal et al., 2011). We may conclude from these combined sensory data that female Targets find the water more distressing initially compared to males. Their enhanced distress calls may be aversive to the Observers and drive release behaviors, while males may be more dependent on sight. While the correlation between reduced latency and diminished distress calls in females suggests this may be the case, these may be two independent findings with no effect on the other. In order to answer this question, future studies could use an opaque Plexiglas divider without a Target and instead play back distress calls to determine if it is sufficient to produce release behavior, especially in females. Finally, these data also imply distress calls alone are not sufficient for the maintenance of empathic behavior (Ben-Ami Bartal et al., 2011). The high frequency calls shown to communicate prosocial information broadly (Panksepp and Burgdorf, 2003; Wohr and Schwarting, 2007; Simola and Brudzynski, 2018) may also drive targeted helping, even in the absence of direct social contact. Moreover, canonically appetitive calls may also be used at different rates in males compared to females. The current understanding of USV is sparse and a lack of baseline control experiments assessing USV also limit our understanding. Further, the distinction of distress from appetitive calls may not be as concise in complex prosocial behaviors, making strong conclusions difficult. For example, the canonical 22 kHz distress call is often observed in the post-ejaculation period in male rats (Bialy et al., 2016), calling the distinct grouping of these calls into question (Bogacki-Rychlik et al., 2022). However, taken together, these experiments indicate sex differences are present in sensory and affective communication, especially early in acquisition when release is less frequent.

A limitation to this evaluation of sensory impacts on targeted helping is the absence of any assessment of olfactory cues on behavioral outcomes. It has been well documented that olfactory signaling plays a large role in prosocial behaviors, as inhibition of olfactory signaling significantly attenuates emotional contagion in rodent models (Fanselow, 1985; Kiyokawa et al., 2009; Takahashi et al., 2013). While this question of the impact of olfaction was outside the scope of this current manuscript, a more thorough evaluation of olfactory signaling within our lab’s targeted helping model is warranted to compare the relative impact of each sensory modality, in both sexes, on behavioral outcomes. An understanding of pheromonal communication may help lend a better understanding of the motivation for prosocial behaviors in Observers, and any possible habituation experienced by Targets during the task.

Since no differences in behavior were seen at any timepoint, correlation of Fos + activity with behavior was combined between males and females. Here, an interesting trend emerged; during EA, significant negative correlations were found between release latency and activity in all cortical areas evaluated, including the AI, PL, IL, ACC, and OFC (Figures 6A–E). However, the PVN of the thalamus was positively correlated with latency (Figure 6F). In contrast, positive correlations were seen in cortical regions, specifically the ACC and PL, during LA (Figures 7D,E). It has been hypothesized that empathy is comprised broadly of two main neurological processes. The first, emotionally driven “bottom-up” subcortical regions, including the thalamus, primarily drive processes that initiate and propagate feelings of shared emotions (Panksepp and Watt, 2011). In contrast, “top-down” cortical circuitry is capable of receiving and regulating the primary emotional information in order to generate appropriate behavioral outputs (see Figure 8C for schematic) (Panksepp and Panksepp, 2013; de Waal and Preston, 2017). While both processes are necessary for prosocial behaviors, the temporal role each plays in targeted helping is still unclear. Our data suggest heightened subcortical, specifically thalamic, activity may contribute to an initial and theoretically unregulated emotional contagion response in early acquisition, hindering release of the conspecific (Figure 6F). However, the cortical regulation of the initial emotional drive for the targeted helping behavior was shown to directly correlate to faster responses, suggesting the “top-down” cortical control is critical even when experiential understanding of the task is low (Figure 6). With progressive trials, higher order cortical regions were shown to be directly related to the significantly attenuated release latency (Figure 7), which may intimate that top-down regulation may become unnecessary as the task becomes more familiar or habitual (Yin and Knowlton, 2006; Panksepp and Panksepp, 2013). Although this is a preliminary analysis and an exploration of the causal effects of the activity of the neural substrates studied are necessary via additional baseline controls and inhibition studies in the future, we may postulate from these data that both cortical and thalamic processes play a role in the measured helping behavior and plasticity of these circuits are seen across time.

When separating males and females for analysis, distinctive sex-specific activity patterns emerged in Observer neural activity. During EA, male and female behaving (BEH) rats had potentiated activity, as measured by the immediate early gene *c-fos*, throughout all of the cortical areas studied, as well as in the PVT and BLA (Figure 8). Increased cortical activity was seen in females during LA. Subcortical areas also had different patterns of expression. Activity in the BLA was significantly potentiated compared to HCC in both male and female BEH rats at similar rates in both timepoints evaluated (Figures 8A,C). The BLA encodes emotionally salient memories, particularly of fear or aversive stimuli (Sun et al., 2020). In contrast, male and female rats exhibited the same pattern of activity in the CeA during EA, but not during LA, as female behaving rats had potentiated CeA neuronal activity compared to female HCC and their male counterparts. Amygdala activity in observer rats generally mirrors that of stressed demonstrators (Knapska et al., 2006), suggesting that the CeA is highly sensitive to the distress of others (Meyza et al., 2017).

Cortical activity diverged between males and females, as total activity of cortical regions in females was significantly higher compared to males during LA (Figure 8C). This elevated Fos expression in females also occurred in HCC females relative to HCC males (Supplementary Figure S1) that is maintained even when correcting for the percent change in Fos + compared to homecage control (HCC) animals (Supplementary Figure S2). Animals with identical experience in the task remained in their homecages on experimental day to act as controls for this analysis. While this control has been used previously (Ben-Ami Bartal et al., 2011, 2021; Cox and Reichel, 2020; Cox et al., 2022), it is possible an additional control setting in which rats placed into the environment in the absence of a conspecific may help elucidate these sex differences. It is unclear why females have significantly higher Fos + cells in multiple cortical regions during LA in the absence of behavioral or ultrasonic vocalization differences. This could represent another divergent sex difference, but further analysis is needed to parse apart the impact of the Fos differences.

A recent study by Ben-Ami Bartal et al. (2021) extensively explored the neural response of rats during a prosocial release task. They also identified similar cortical areas were potentiated during the task compared to baseline levels. A unique aspect of their study focused on neural substrates that varied depending on prosocial intent. For example, the AI, ventral and lateral OFC, and ACC were all potentiated in rats that released both ingroup and outgroup conspecifics, indicating these regions respond to a distressed rat regardless of social context (Ben-Ami Bartal et al., 2021). In contrast, the PL and medial OFC were potentiated during targeted helping of ingroup, compared to outgroup, animals and baseline. The authors concluded that these regions played a specific role in the prosocial response toward ingroup animals and not to a trapped animal or social exposure *per se* (Ben-Ami Bartal et al., 2021). In our studies, the distressed Targets were always the Observers’ cagemates, meaning we did not evaluate for differences between social group. However, our results seem to corroborate the importance of these cortical substrates in targeted helping even when direct social contact is not possible.

Future studies should also focus on additional substrates, such as the Nucleus Accumbens (NAc), instrumental in motivational and reward processing. More meticulous assessment of the impact of the motivation and learning process behind this targeted helping task via the NAc could aid in untangling the prosocial and the habituation of the task across time.

In conclusion, this set of experiments sought to elucidate the role of sex on targeted helping, along with sensory, affective, and neural components that may contribute to helping behavior. We believe that a convergent sex effect may be present in targeted helping, in which males and females exhibit similar behavioral outcomes, but multiple and disparate variables work together to generate the behavior (Hernandez-Lallement et al., 2022). Additionally, sex differences previously understood to be canon are likely dependent on contextual cues and task type (Langford et al., 2006). Nuances of housing (Yamagishi et al., 2020), estrous cycle (Mora et al., 1996; Mikosz et al., 2015), familiarity (Ben-Ami Bartal et al., 2011, 2021), and chronicity of the task (Yin and Knowlton, 2006) must be considered when any affective or physiological variable is being studied as it pertains to helping behaviors, as evidence indicates they may have substantial impact on behavioral outcomes. Overall, we believe these initial studies lay a groundwork for future studies to explore unique social, sensory, and neurobiological variables that drive empathic behavior in both males and females.

## Data availability statement

The raw data supporting the conclusions of this article will be made available by the authors, without undue reservation.

## Ethics statement

The animal study was approved by Institutional Animal Care and Use Committee (IACUC) of the Medical University of South Carolina. The study was conducted in accordance with the local legislation and institutional requirements.

## Author contributions

SC: Conceptualization, Data curation, Formal analysis, Funding acquisition, Investigation, Writing – original draft, Writing – review & editing. BB: Data curation, Writing – review & editing. SW: Data curation, Visualization, Writing – review & editing. SB: Data curation, Investigation, Writing – review & editing. AK: Data curation, Investigation, Project administration, Resources, Writing – review & editing. CR: Conceptualization, Formal analysis, Funding acquisition, Investigation, Methodology, Supervision, Visualization, Writing – review & editing.
