# Supplementary material for: Neuronal, affective, and sensory correlates of targeted helping behavior in male and female Sprague Dawley rats
Source: Front Behav Neurosci. 2024 Apr 10;18:1384578. doi: 10.3389/fnbeh.2024.1384578 (PMC11041374; doi:10.3389/fnbeh.2024.1384578)
Supplement: Supplementary file 1 [file Data_Sheet_1.DOCX]

**SUPPLEMENTAL FIGURES**


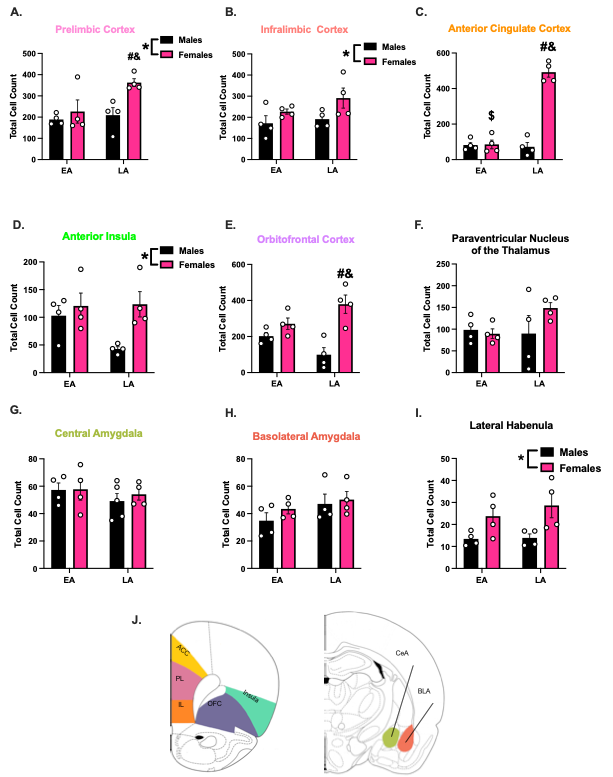


**Supplemental Figure 2**. **Sex Differences in Homecage Controls within Neural Substrates of Interest.**

Groups of male and female rats (n=4/sex) acting as homecage controls (HCC) were sacrificed at one of three-timepoints; early acquisition (EA), late acquisition (LA). Total Fos+ cells for HCC rats were counted in each region of interest and compared at each timepoint. A 2-way ANOVA was calculated, with the variables being sex (males vs. females) and time (EA vs. LA). Post hocs that were reported specifically focused on comparing across sex at the same timepoint and within sex across all timepoints. **A**) Within the prelimbic cortex, the analysis revealed significant effects of both time [F (1,12) = 5.15, *p* = 0.042] and sex [F (1,12) = 7.66, *p* = 0.017]. Post hoc comparisons of note showed differences in total Fos+ cells between LA males and females (*p* = 0.042) and EA males and LA females (*p* = 0.023). **B**) A significant sex effect was seen in the infralimbic cortex (IL) [F (1,12) = 5.89, *p* = 0.032]. **C**) Within the anterior cingulate cortex, main effects of sex [F (1,12) = 79.93, *p*<0.0001], time [F (1,12) = 70.21, *p*<0.001], along with a sex x time interaction [F (1,12) = 77.26, *p*<0.0001] were significant. Relevant post hocs concluded Fos+ cells for females at the LA timepoint were significantly higher than males and females at EA, as well as LA males (p<0.0001). **D**) The 2-way ANOVA in the anterior insula revealed a significant effect of sex [F (1,12) = 6.84, *p* = 0.023], but neither a main effect of time nor an interaction was found. **E**) Analysis in the orbitofrontal cortex revealed main effects of sex [F (1,12) = 21.94, *p*=0.0005] and a sex x time interaction [F (1,12) = 8.063, *p*=0.015]. Post hoc analyses showed females at the LA timepoint had significantly higher Fos+ cell counts compared to EA (p=0.03) and LA (p=0.0011) males. **F**) Neither a main effect of sex nor a main effect of time was observed in the analysis for the paraventricular nucleus of the thalamus. **G**) Similarly, no effect was seen in the analysis of the central amygdala, **H**) or the basolateral amygdala. **I**) Only a main effect of sex was seen in the lateral habenula (LHb) [F (1,12) = 11.20, *p* = 0.0058]. **J)** Brain atlas representation of the regions of interest in the Fos analysis.

* p<0.05

**#**Significant difference from same timepoint of other sex, p<0.05

&Significant difference from early acquisition of other sex, p<0.05

**$**Significant difference from late acquisition of own sex, p<0.05

**Supplemental Figure 3. Percent Change Differences in Fos+ cells across Neural Regions of Interest.**

Male and female rats seem to exhibit a similar pattern of behavior during the task during EA and LA (**Figure 2**), but there is a sex difference inherent in the baseline activity of homecage control (HCC) animals between sex in many of the regions examined (**Supplemental Figure 2**), suggesting a quantitative sex effect. This effect may be producing the convergent effect seen in behavior, in which males and females exhibit similar targeted helping, but the underlying process mediating it differs^59^. Therefore, to better compare Fos levels between sexes, we converted the total Fos+ cell counts into a percent of HCC for each group using the following formula: $\%HCC= \frac{{Fos}_{BEH}}{{Fos}_{HCC}}$ , with Fos+_EMP_ being the total Fos+ cell count in the BEH group, and Fos+_HCC_ the total in the respective HCC animals. Values are set to 100% of HCC. That way, the overall sex differences in basal neural activity can be accounted for, and the sexes can be directly compared between the three timepoints. For each neural substrate of interest, mixed effect analysis was performed, and where appropriate, post hocs comparing each timepoint (EA vs. LA) were compared within sex to understand if the trend in activity across time in each region was different in males compared to females. **A**) Within the prelimbic cortex, no effect of sex or time was found. **B**) The infralimbic cortex analysis showed a main effect of time [F (1,16) = 9.259, *p* = 0.0078], as well as a time x sex interaction [F (1,16) = 7.539, *p* = 0.00144]. Specifically, LA males had an attenuated % change in Fos+ cells compared to both EA males (*p* = 0.0008), with no changes within females. **C**) No significance was observed in the analysis of the Anterior Cingulate. **D**) An approximate two-fold increase in Fos+ cells compared to HCC was seen within the insula across all groups, but no main effects of time or sex were noted. **E**) The mixed effect analysis of the orbitofrontal cortex showed a main effect of sex [F (1,8) = 14.68, *p* = 0.0050]. **F**) Within the paraventricular nucleus of the thalamus, there was nothing significant revealed in the analysis. **G**) A main effect time [F (1,6) = 14.28, *p* = 0.0092] was found in the central amygdala only. **H**) No effects were found in the basolateral amygdala **I**) In the lateral habenula, while there was a trend for a main effect of sex (p=0.074), it did not reach significance. **J)** A heatmap representation of the mean %change in Fos+ cell activity of each brain region at each timepoint for both males and females.

*Significant change from HCC, p<0.05

#Significant difference from EA of own sex, p<0.05

&Significant difference from LA, p<0.05

^Main effect of sex, p<0.05
